# Predictors Associated With Knowledge and Practice of Helminthic Infection Prevention Among Rural School-Aged Children's Parents in Bangladesh: A Cross-Sectional Study

**DOI:** 10.3389/fpubh.2020.00484

**Published:** 2020-09-04

**Authors:** Md. Safaet Hossain Sujan, Md. Saiful Islam, Shabnam Naher, Rajon Banik, David Gozal

**Affiliations:** ^1^Department of Public Health and Informatics, Jahangirnagar University, Dhaka, Bangladesh; ^2^Youth Research Association, Dhaka, Bangladesh; ^3^Department of Child Health and the Child Health Research Institute, The University of Missouri School of Medicine, Columbia, MO, United States

**Keywords:** helminths, infection prevention, school-age children, rural area, Bangladesh

## Abstract

**Background:** Parasitic infection has become a major public health concern in light of its increasing prevalence in developing countries, particularly in rural settings. Helminthic infections disproportionately affect children, and therefore appropriate parental knowledge and practical approaches to transmission prevention are essential. The objective of this study was to evaluate the level of knowledge and implementation measures aimed at preventing helminthic infection among rural school-aged children's parents in Bangladesh.

**Methods:** A cross-sectional survey was conducted among the parents of 449 rural school-aged children residing in 17 villages of the Cumilla district in Bangladesh. Participants completed the survey examining socio-demographic variables as well as questions regarding knowledge and practice of helminthic infection prevention (HIP).

**Results:** Local knowledge about children's HIP was inadequate among their parents (81.5%). Furthermore, actual implementation of HIP measures was poor (42.1%). Knowledge of HIP was significantly associated with parental religion, education, occupation, number of family members, family income, housing conditions, and sanitation system. In addition, practice of HIP was significantly associated with the aforementioned factors, as well as with water source and knowledge of HIP.

**Conclusion:** Lack of concern about parasitic infection among children's parents and their untoward effects on children's health is pervasive in rural parental settings, along with ignorance on helminthic transmission and its prevention. Public education programs aimed at addressing these glaring HIP awareness deficits are needed in Bangladesh.

## Introduction

Helminthiasis is a parasitic worm infection that primarily invades the intestinal tract in humans, but can extend to almost any other internal organ. Humans are mostly infected by Soil-Transmitted Helminthiasis (STH), caused by a group of parasites that includes roundworm (*Ascaris lambricoides*), whipworm (*Trichuris trichiura*) and hookworm (*Ancylostoma duodenale*) (1). Parasitic infection has progressively become a major global public health issue, and imposes major socio-economic burden (2). The major at-risk groups are children of pre-school age (pre-SAC) or school-age (SAC), and women of childbearing age.

According to the World Health Organization (WHO) estimates, more than 1.5 billion people (~24%of the world population), are infected with soil-transmitted helminth infections worldwide, particularly in tropical and subtropical areas (3). Among them, more than 267 million pre-SAC (1–5) and over 568 million SAC (6–11) live in areas where these parasites are intensively transmitted, and are in need of treatment and preventive interventions. STH infected children are nutritionally and physically impaired, and suffer from a large number of health issues, including intestinal manifestations, malnutrition, general malaise and weakness, and impaired somatic and intellectual growth, with very high parasite burden potentially leading to intestinal obstruction (3, 4).

Bangladesh's 64 districts are endemic for STH (5), and a national parasitology survey in 2010 reported that 79.8% of SAC were infected with one or more helminthic species (6). The most neglected tropical disease, human trichuriasis is particularly prevalent among children who are dwelling in places where the sanitation system is inadequate (7). Children residing in rural Bangladesh are most likely to be affected, where soil-transmitted helminthic infection (STHI) and aquatic infection (AI) are inordinately common in light of the large proportion of the population that walks barefoot, and consumes inappropriately processed water or food. The high prevalence rate of the parasitic infection is usually correlated with poverty, poor environmental and personal hygiene, and insufficient health services (4). However, to enable effective STHI control measures, awareness to both the disease, and its prevention are critical. The present study was conducted to evaluate the current status of knowledge and implementation of helminthic infection prevention (HIP) among parents of SAC residing in rural regions of Cumilla, Bangladesh, and to identify potential risk factors that may contribute to the high prevalence of STHI in this population. The knowledge gained from the survey should be valuable for designing and developing helminthic control initiatives in such rural regions.

## Methods

### Participants and Procedures

**Figure 1:**
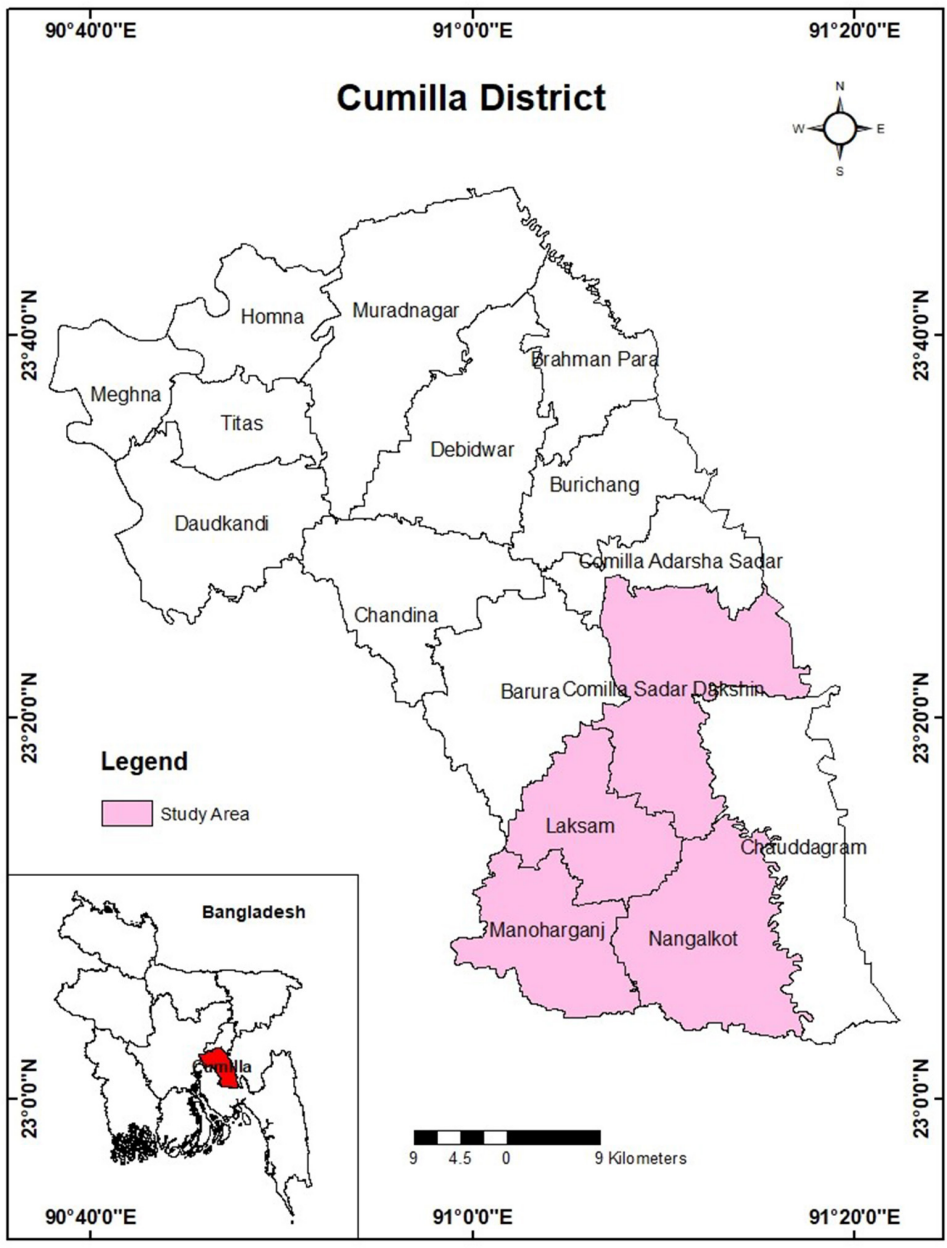
Study area.

A descriptive cross-sectional study was conducted among the parents of 449 school-aged (6–11 years) children residing in 17 villages of four Upazila (Cumilla Sadar Dakhsin, Laksam, Monohorgonj, and Nangolkoat; Figure 1). A semi-structured questionnaire was used to conduct face-to-face interviews during the study period. A purposive sampling technique was applied to draw the sample from the population. Only one parent participated in the study from a single household. Data were collected from April 2019 to September 2019. The inclusion criteria to participate in the study were being a school-aged (6–11) children's father or mother, and dwelling in the pre-determined rural area. We excluded mentally challenged parents. Data collection was performed by approaching a house, and then skipping 2–3 houses, and then try another house asking to interview the household head (HH). If the HH was not present, no further attempts were made in that specific residence.

### Measures

A pre-tested questionnaire containing informed consent and questions divided into 3 sections was used to conduct the study using face-to-face interviews. The participants were asked to allocate 20–30 min of their time for completion of the questionnaire. Section-Introduction contained socio-demographic variables including age, gender, religion, educational qualifications, number of members of the family, occupation of the respondents, monthly family income, housing conditions, sanitation system, and drinking water source. Monthly family income was categorized into three classes based on monthly family income: lower-class (<10,000 BDT), middle-class (10,000–20,000 BDT), and upper-class (more than 20,000 BDT) (8, 9). Section-Methods consisted of questions aiming to assess the knowledge regarding helminthic infection and its health effects. Section-Results consisted of questions to assess the practice of helminthic infection prevention as well as personal hygiene and sanitation. Knowledge regarding STHI and prevention approaches were categorized using the approach described by Mohd and Itrat (10). Section-Methods consisted of 12 multiple choice questions whereas Result included 9 multiple questions. For each correct response a score of one “1” was given and a zero “0” score was assigned to each wrong response. Here, we defined “knowledge” as familiarity with, or comprehension of STHI, while “prevention practice” consisted of the steps taken to avoid STHI. Knowledge was scored as adequate knowledge (≥8/12) and inadequate knowledge (<8/12). Target population practice was measured according to the previous study [i.e., Islam et al. (11)]. Preventive practice scores were also labeled as good (≥6/9) and poor (<6/9).

#### Statistical Analysis

The data were analyzed using Statistical Software (SPSS) version 25.0. Descriptive statistics (frequencies, percentages, etc.) and cross-tabulations and chi-square tests were performed to determine significant relationships between categorical dependent and independent variables.

#### Ethical Considerations

All procedures were performed in accordance with the Institutional Research Ethics and the Code of Ethics of the World Medical Association (Declaration of Helsinki) for experiments involving humans. Formal ethical approval was granted from the respective authorities. Verbal informed consent (from illiterate) or written informed (from literate) consent was obtained from all respondents prior to data collection. All the participants were provided with information concerning the purpose of the study prior to data collection and the objective of the research was explained. The confidentiality of their information, the nature of the study, the decision to take part in the study, and the right to withdraw their data at any time from the study, were all outlined in the consent form. They were given enough time to go through the study details and had the opportunity to ask questions. After they agreed to participate in the study, they were requested to sign the informed consent form.

## Results

The participants of the study comprised a total of 449 rural parents including 44.3% males and 55.7% females, and their ages ranged from 25 to 39 years. Demographic data are presented in Table 1. Almost three-fourths of the respondents (70.2%) were illiterate, 27.4% of respondents had primary education, and the remainder (2.4%) had secondary or higher education. The majority of the respondents (94.0%) were Muslim and only 6.0% of respondents were Hindus. Housekeeping (51.4%) and farming (34.1%) comprised the majority of the occupations, with the rest either being employed or having a small business. More than five members in the family were reported among 82.0% of the responders. According to monthly family income, 82.0% were in the low-class, 17.6% of respondents were in the middle-class and only 0.4% of respondents were in the upper-class. The majority of the respondents (72.2%) dwelled in a house made of tin with the floor being soil, while 17.4% live in raw clay houses, and only 10.5% of respondents lived in pucca houses. In 79.1% of the families, the sanitation system consisted of pit-latrine such that the defecation materials would be easily mixed with water and soil. Only 2.4% of respondents indicated that they defecated in open spaces, and only 18.5% of the houses included latrine with septic tank or connected with sewerage network system. Among respondents, 81.5% exhibited poor knowledge regarding STHI, while only 18.5% had adequate knowledge on STHI including transmission and symptoms. The majority of the respondents (57.9%) engaged in poor practices toward HIP.

**Table 1:** Distribution of variables, and association with knowledge and practice among rural school-aged children's parents.

**Variables**			**Knowledge**							**Practice**						
	**Total** ***N*** **=** **449**		**Adequate**		**Inadequate**		**χ^**2**^**	**df**	***p*-value**	**Good**		**Poor**		**χ^**2**^**	**df**	***p*-value**
	***N***	**(%)**	***N***	**(%)**	***N***	**(%)**				***N***	**(%)**	***N***	**(%)**			
**Gender**																
Male	199	(44.3)	29	(34.9)	170	(46.4)	3.63	1	0.057	76	(40.2)	123	(47.3)	2.23	1	0.135
Female	250	(55.7)	54	(65.1)	196	(53.6)				113	(59.8)	137	(52.7)			
**Age**																
25–29	22	(4.9)	5	(6.0)	17	(4.6)	3.25	2	0.197	12	(6.3)	10	(3.8)	3.33	2	0.189
30–34	414	(92.2)	78	(94.0)	336	(91.8)				174	(92.1)	240	(92.3)			
35–39	13	(2.9)	0	(0.0)	13	(3.6)				3	(1.6)	10	(3.8)			
**Religion**																
Muslim	422	(94.0)	74	(89.2)	348	(95.1)	4.20	1	**0.04**	170	(89.9)	252	(96.9)	9.42	1	**0.002**
Hindu	27	(6.0)	9	(10.8)	18	(4.9)				19	(10.1)	8	(3.1)			
**Educational qualification**																
Illiterate	315	(70.2)	33	(39.8)	282	(77.0)	60.69	2	** <0.001**	93	(49.2)	222	(85.4)	68.73	2	** <0.001**
Primary	123	(27.4)	41	(49.4)	82	(22.4)				89	(47.1)	34	(13.1)			
Secondary	11	(2.4)	9	(10.8)	2	(0.5)				7	(3.7)	4	(1.5)			
**Occupation**																
Farming	153	(34.1)	15	(18.1)	138	(37.7)	31.48	3	** <0.001**	47	(24.9)	106	(40.8)	27.99	3	** <0.001**
Job	42	(9.4)	20	(24.1)	22	(6.0)				30	(15.9)	12	(4.6)			
Business	23	(5.1)	5	(6.0)	18	(4.9)				15	(7.9)	8	(3.1)			
Housewife	231	(51.4)	43	(51.8)	188	(51.4)				97	(51.3)	134	(51.5)			
**Family members**																
<5	368	(82.0)	78	(94.0)	290	(79.2)	9.94	1	**0.002**	171	(90.5)	197	(75.8)	16.01	1	** <0.001**
≥5	81	(18.0)	5	(6.0)	76	(20.8)				18	(9.5)	63	(24.2)			
**Monthly family income**																
Lower class	368	(82.0)	58	(69.9)	310	(84.7)	16.56	2	** <0.001**	142	(75.1)	226	(86.9)	13.12	2	**0.001**
Middle class	79	(17.6)	23	(27.7)	56	(15.3)				47	(24.9)	32	(12.3)			
Upper class	2	(0.4)	2	(2.4)	0	(0.0)				0	(0.0)	2	(0.8)			
**Housing condition**																
Raw Clay House	78	(17.4)	8	(9.6)	70	(19.1)	29.50	2	** <0.001**	11	(5.8)	67	(25.8)	45.84	2	** <0.001**
Tin Shed	324	(72.2)	53	(63.9)	271	(74.0)				143	(75.7)	181	(69.6)			
Building	47	(10.5)	22	(26.5)	25	(6.8)				35	(18.5)	12	(4.6)			
**Sanitation system**																
Open space	11	(2.4)	0	(0.0)	11	(3.0)	31.99	2	** <0.001**	1	(0.5)	10	(3.8)	25.63	2	** <0.001**
Raw Closet	355	(79.1)	50	(60.2)	305	(83.3)				134	(70.9)	221	(85.0)			
Building	83	(18.5)	33	(39.8)	50	(13.7)				54	(28.6)	29	(11.2)			
**Knowledge**																
Adequate	83	(18.5)	83	(100.0)	0	(0.0)				52	(27.5)	31	(11.9)	17.65	1	** <0.001**
Inadequate	366	(81.5)	0	(0.0)	366	(100.0)				137	(72.5)	229	(88.1)			
**Practice**																
Good	189	(42.1)	52	(62.7)	137	(37.4)	17.65	1	** <0.001**	189	(100.0)	0	(0.0)			
Poor	260	(57.9)	31	(37.3)	229	(62.6)				0	(0.0)	260	(100.0)			

*Bold values indicate significant variables at 5% significance level*.

The majority of the participants (78.8%) knew the term STHI and had learned it from a doctor (58.2%) (Table 2). Regarding the reasons for helminthic infection, 35.0% participants reported using latrines without protective footwear, and 44.1% reported that no knowledge of the source of infection. Regarding problems among children suffering from helminthic infection, 33.9% reported reluctance to study or to conduct other academic activities, and 44.3% reported no awareness to any problems; however, 35.4% of the participants reported that helminth infected children suffered from malnutrition, and 33.4% reported that their children suffered from stomach aches and rectal irritation.

**Table 2:** Knowledge, practice and gender differences among rural school-aged children's parents (*N* = 449).

**Variable**				**Gender**						
		**Total** ***N*** **=** **449**		**Male**		**Female**		**χ^**2**^**	**df**	***p-*value**
		***N***	**(%)**	***N***	**(%)**	***N***	**(%)**			
**STHI knowledge**										
Knowledge	Adequate	83	(18.5)	29	(14.6)	54	(21.6)	3.63	1	0.057
	Inadequate	366	(81.5)	170	(85.4)	196	(78.4)			
**Practice**										
Parents activities after child affected by helminths infection	Consult doctor	245	(54.6)	115	(57.8)	130	(52.0)	12.11	3	**0.007**
	Drink water of lordship	87	(19.4)	26	(13.1)	61	(24.4)			
	Take advice from tantric	6	(1.3)	1	(.5)	5	(2.0)			
	Did nothing	111	(24.7)	57	(28.6)	54	(21.6)			
Advise children to wash their hands before and after eating	Yes	201	(44.8)	76	(38.2)	125	(50.0)	6.25	1	**0.012**
	No	248	(55.2)	123	(61.8)	125	(50.0)			
Children use the toilet without sandals	Yes	199	(44.3)	81	(40.7)	118	(47.2)	1.90	1	0.169
	No	250	(55.7)	118	(59.3)	132	(52.8)			
Children wash their hands with soap after using the toilet	Yes	158	(35.2)	72	(36.2)	86	(34.4)	0.15	1	0.695
	No	291	(64.8)	127	(63.8)	164	(65.6)			
Source of the drinking water of family	Pond	34	(7.6)	16	(8.0)	18	(7.2)	1.37	2	0.504
	Deep tube-well	409	(91.1)	179	(89.9)	230	(92.0)			
	Supply water	6	(1.3)	4	(2.0)	2	(0.8)			
Habit of eating enough boiled food	Yes	376	(83.7)	161	(80.9)	215	(86.0)	2.11	1	0.146
	No	73	(16.3)	38	(19.1)	35	(14.0)			
Time of washing fruits and vegetables	Before cutting	109	(24.3)	47	(23.6)	62	(24.8)	0.09	1	0.772
	After cutting	340	(75.7)	152	(76.4)	188	(75.2)			
Children eat fruits after proper washing	Yes	253	(56.3)	108	(54.3)	145	(58.0)	0.63	1	0.429
	No	196	(43.7)	91	(45.7)	105	(42.0)			
Gave children anti-helminths drugs at least two times in a year	Yes	271	(60.5)	118	(59.6)	153	(61.2)	0.12	1	0.730
	No	177	(39.5)	80	(40.4)	97	(38.8)			

*Bold values indicate significant variables at 5% significance level*.

In 24.7% of respondents, parents did not intervene when their child presented with an STHI, while 54.6% consulted the village physician. Approximately 44.8% of respondents had their children wash hands before and after eating, 64.8% of children did not wash their hands with soap after defecation, and 55.7% of the children used toilets without sandals; furthermore, 91.1% drank deep tube well-water, while 16.3% ate unboiled food, and 24.3% did not wash fruits and vegetables before consumption; 43.7% indicated that their children did not wash fruits before eating. Approximately 60.5% of respondents gave their children anti-helminthic drugs at least two times a year, while the remaining 39.5% of respondents did not use any anti-helminthic medications.

As shown in Table 1, knowledge of HIP was significantly associated with religion (χ^2^= 4.2, *df* = 1, *p* = 0.04), education (χ^2^= 60.69, *df* = 2, *p* < 0.001), occupation (χ^2^= 31.48, *df* = 3, *p* < 0.001), number family members per household (χ^2^= 9.94, *df* = 1, *p* = 0.002), family income (χ^2^= 16.56, *df* = 2, *p* < 0.001), housing condition (χ^2^= 29.5, *df* = 2, *p* < 0.001), and sanitation system (χ^2^= 31.99, *df* = 2, *p* < 0.001). The practice of HIP was significantly associated with religion (χ^2^= 9.42, *df* = 1, *p* = 0.002), education (χ^2^= 68.73, *df* = 2, *p* < 0.001), occupation (χ^2^= 27.99, *df* = 3, *p* < 0.001), number of family members per household (χ^2^= 16.01, *df* = 1, *p* < 0.001), family income (χ^2^= 13.12, *df* = 2, *p* < 0.001), housing condition (χ^2^= 45.84, *df* = 2, *p* < 0.001), sanitation system (χ^2^= 25.63, *df* = 2, *p* < 0.001), water source (χ^2^= 5.96, *df* = 2, *p* = 0.05), and HIP knowledge (χ^2^= 17.65, *df* = 1, *p* < 0.001).

## Discussion

STH is still one of Bangladesh's most significant public health issues. Here, we show that a majority of residents in rural Bangladesh is unaware of the effects of STHI on health, and such knowledge gaps appear to be equally distributed among men and women. This study is the first to evaluate parents of school-aged children regarding knowledge and practice in the field of helminthiasis. As can be observed from the rigorous efforts to control the disease, STH is still highly prevalent among children in several areas of the country. Several researchers reported a greater prevalence of STH in various regions of Bangladesh (12). A significant number of studies carried out in several tropical countries where STH is endemic, such as Malaysia (13), China (14), Philipines (15) and Ivory Coast (2) has yielded similar findings to our current study. There are massive gaps between data about knowledge on the effects of the disease, and the residents' opinions on disease prevention. Lack of knowledge about the disease, who may be at danger for the disease, how and when to target all at-risk populations, such as the need to target symptomless carriers and misrepresentation of the drug about to be administered by the community are all extremely frequent (5). A significant number of respondents believed that worms were spread from eating sweet foods. This seems to be a common misconception in Bangladesh regarding worms (16).

As reported by the Directorate General of Health Services, Ministry of Health and Family Welfare, Bangladesh, the prevalence of any STHI among school-age children was 40% compared to 80% in rural areas before school deworming programs were initiated (17). The Global Health Initiative (GHI) of USAID intends to minimize the incidence of seven neglected tropical diseases (NTDs) by at least 50 percent among 70 percent or more of the population affected. To successfully achieve such goal globally, nationwide deworming systems use a Mass Drug Administration (MDA) method to provide deworming pills for all children of school age. Programs that routinely distribute deworming pills to all school-age children are not only treating the infected people, but also helping to reduce the community burden. When successfully implemented, such approaches lead to lower total worm loads, and reduce re-infection rates. The medications used to treat the most serious STH infections are safe, effective, cost-effective and easy to administer (18). Before launching the program, the prevalence of infected SAC was 79.8% (18). However, a recent study conducted in a rural area found the prevalence had declined to 39.2% (19). In a previous study (5) among SAC, 65% of parents had never heard about intestinal worms, and 33.8% of parents were ignorant of the ill effects and consequences of the disease. In the present study, awareness among participants of the signs, modes of transmission and preventive measures was poor, and exhibited educational level and socioeconomic status dependencies. Furthermore, false beliefs regarding the mode of transmission were pervasive among participants with substantial unawareness of the risks of unhygienic toilets, barefooted toilet use, lack of handwashing hands and consuming contaminated food, all of which constitute major obstacles to any control initiatives (13).

Furthermore, the current study shows that SAC's parents are unaware that STH is transmitted via contaminated soil and unhygienic practices, along with, but not limited to, toilet use, absence of handwashing and often defecation. Parents should be educated and encouraged to facilitate good hygiene practices such as appropriate use of latrines to avoid contamination of the surroundings, and handwashing among SAC. A study recently uncovered that minimal attention and awareness to soil as a reason for contamination, and recommended performance of preventive chemotherapy, education programs, and group use of sanitary toilets to lessen STHI (20).

Thus, to enable successful campaigns aimed at STHI prevention among SAC, there is a need to provide not only specific information about STHI, i.e., incrementing “knowledge,” but also explicitly address issues such as consistent use of sanitary toilets to prevent contamination of the environment, as recently reported by the World Health Organization (21). In Bangladesh, achieving the national STHI elimination target date of 2025 will require multi-sectoral collaboration and integration of the control strategies that have already demonstrated efficacy. Health and hygiene education must be taught in schools so that SAC can easily learn about them and adopt some of those protective measures. In parallel, installation of adequate and proper sanitation systems needs to be put in place by government and other public interest agencies. In addition, the negative impacts of helminthic manifestation on SAC should be well-understood by their parents and translated into their daily lives. Further steps in this program will be to address misconceptions with educational campaigns, train teachers to administer deworming medication, implement systematic programs for improved water and sanitation facilities and appropriate policy of mass treatment in all target populations. All of these measures must be implemented synchronously in order to achieve effective control of STHI, and will require cooperation from the local communities, health workers, teachers, and increased public support in the form of both “knowledge” and “prevention practice.” The multifactorial elements associated with poor STHI knowledge and HIP practices as identified in the current study further reinforce the urgent need for multidimensional efforts to contain and reduce the unacceptably high STHI rates in Bangladeshi rural SAC, and prevent deleterious short-term and long-term consequences.

### Study Strengths, Limitations, and Recommendations

To the best of our knowledge, this study is the first to evaluate parents of school-aged children in the field of helminthiasis prevention. The targeted cohort resided in one of the most populated districts with poverty, poor sanitation system, and illiteracy. However, this was a cross-sectional study, such that any inferences on causality are not possible. Secondly, our study was confined to a single rural region of Bangladesh; hence, findings may vary in other regions. Finally, we did not perform specific soil sampling and STHI detection in the children. Stool sampling of the children would be highly desirable to evaluate the prevalence of STHI along with subsequent longitudinal monitoring and intervention outcomes.

## Conclusion

In summary, parents of SAC in rural Bangladesh settings demonstrate inordinately high levels of inadequate knowledge regarding STHI in children and their adverse health effects. Furthermore, this lack of knowledge is further compounded by existing poor practices regarding the prevention of helminthic infections. There is little doubt that in order to achieve eradication of STHI in the majority of the SAC population, effective health education and promotion campaigns targeting parents and the public in general, will be essential and need to incorporate not only “knowledge-based elements” but also address many of the factors associated with poor prevention as identified herein.

## Data Availability Statement

The original contributions presented in the study are included in the article/supplementary material, further inquiries can be directed to the corresponding author/s.

## Ethics Statement

The studies involving human participants were reviewed and approved by the Ethical Board of Jahangirnagar University, Savar, Dhaka, Bangladesh. The patients/participants provided their written informed consent to participate in this study.

## Author Contributions

SN designed the study. MS and MI performed data collection, data analysis, interpretation and contributed to the manuscript write up. Then SN, RB, and DG edited the manuscript and contributed to interpretation of results. All authors contributed to the manuscript. Finally, all authors read and approved the final manuscript.

## Conflict of Interest

The authors declare that the research was conducted in the absence of any commercial or financial relationships that could be construed as a potential conflict of interest.
